# Comparative DIA Proteomics of Neuroblastoma: Laser-Microdissected FFPE Tumors Versus Cell Lines

**DOI:** 10.3390/biology15201839

**Published:** 2026-10-14

**Authors:** Akihiro Nishimura, Tomoko Fujikawa, Shotaro Inoue, Nobuyuki Yamamoto, Makiko Yoshida, Tetsuya Fukuda, Yasuhiko Bando, Kandai Nozu, Daiichiro Hasegawa, Aya Yoshioka, Hiroyuki Sonoda, Toshihide Nishimura, Akihiro Tamura

**Affiliations:** 1 Department of Pediatrics, Kobe University Graduate School of Medicine, Kobe 650-0017, Japan; nishimura.f.wdw@cs.u-ryukyu.ac.jp (A.N.); tomoko14@med.kobe-u.ac.jp (T.F.); stinoue@med.kobe-u.ac.jp (S.I.); nyama@med.kobe-u.ac.jp (N.Y.); nozu@med.kobe-u.ac.jp (K.N.); 2 Department of Child Health and Welfare (Pediatrics), Graduate School of Medicine, University of the Ryukyus, Okinawa 901-2222, Japan; 3 Department of Pathology, Hyogo Prefectural Kobe Children’s Hospital, Kobe 650-0047, Japan; mkyoshida_kch@hp.pref.hyogo.jp; 4 Department of Research and Development, Biosys Technologies, Inc., Tokyo 153-8904, Japan; t-fukuda@biosys-tech.co.jp (T.F.); y-bando@biosys-tech.co.jp (Y.B.); 5 Department of Hematology and Oncology, Children’s Cancer Center, Hyogo Prefectural Kobe Children’s Hospital, Kobe 650-0047, Japan; hasegawa_kch@hp.pref.hyogo.jp; 6 Research Division, JCR Pharmaceuticals Co., Ltd., Kobe 659-0021, Japan; yoshioka-a@jp.jcrpharm.com (A.Y.); sonoda-h@jp.jcrpharm.com (H.S.); 7 Department of Chest Surgery, St. Marianna University School of Medicine, Kawasaki 216-8511, Japan; t-nisimura@marianna-u.ac.jp

**Keywords:** neuroblastoma, formalin-fixed paraffin-embedded (FFPE), laser microdissection, data-independent acquisition proteomics, nano-LC/MS, cell lines, comparative proteomics, tumor microenvironment, pediatric cancer

## Abstract

Formalin-fixed, paraffin-embedded (FFPE) tumor specimens are widely available and represent a valuable resource for retrospective cancer research; however, chemical cross-linking has historically limited their application in proteomic studies. In this study, we developed a proteomic workflow for laser-microdissected FFPE neuroblastoma tissues and evaluated their biological characteristics in comparison with established neuroblastoma cell lines. Tumor regions were isolated from FFPE sections by laser microdissection, and protein profiles were analyzed using data-independent acquisition mass spectrometry. Seven high-risk neuroblastoma FFPE samples and four neuroblastoma cell lines were examined. A total of 4511 proteins were identified in FFPE tissues and 6812 proteins in cell lines, with 4311 proteins shared between both systems. After quality filtering, 2186 proteins were retained for downstream quantitative analyses. Principal component analysis and hierarchical clustering demonstrated clear separation between FFPE tissues and cell lines despite a strong overall correlation in mean protein abundance across the quantified proteome. Differential abundance and functional enrichment analyses revealed distinct proteomic features between the two systems. Proteins enriched in FFPE tissues were associated with extracellular vesicles, the extracellular space, the cornified envelope, intermediate filament organization and cell junctions. In contrast, proteins enriched in neuroblastoma cell lines were associated with RNA binding, translation, rRNA processing, oxidative phosphorylation, and glycolysis/gluconeogenesis, reflecting enhanced biosynthetic and metabolic activity. Together, these findings demonstrate that FFPE tissues and neuroblastoma cell lines capture complementary aspects of neuroblastoma biology, supporting their combined use as a comprehensive framework for biomarker discovery and translational research in pediatric oncology.

## Simple Summary

Neuroblastoma is a childhood cancer that often has a poor prognosis despite intensive treatment. Researchers frequently use cancer cells grown in the laboratory to study this disease, but it remains unclear how well these models represent tumors in patients. Hospitals routinely preserve tumor samples as formalin-fixed, paraffin-embedded (FFPE) tissues, which are linked to clinical information and stored long-term. If proteins can be reliably measured from these archived samples, they could provide valuable insights into real tumor biology without requiring new tissue collection. In this study, we compared protein profiles between laser-microdissected FFPE neuroblastoma tissues and established neuroblastoma cell lines using mass spectrometry–based proteomics. We found that FFPE tissues retain extracellular, structural, and microenvironment-associated features present in native tumors, whereas cell lines predominantly reflect proliferative, biosynthetic, and metabolic programs characteristic of in vitro growth. Although substantial overlap was observed between the two systems, each captured distinct aspects of neuroblastoma biology. These findings support the complementary use of FFPE tissues and cell-line models and highlight the value of archived clinical specimens for biomarker discovery and translational research in pediatric oncology.

## 1. Introduction

Formalin-fixed, paraffin-embedded (FFPE) tissues are widely available and clinically annotated specimens that serve as an indispensable resource for translational pathology, particularly in the era of precision oncology. Their long-term stability and direct linkage to detailed clinicopathological information enable large-scale retrospective analyses that are otherwise difficult to perform using fresh or frozen materials. Recent methodological advances in laser microdissection (LMD), optimized SDS-based protein extraction, and nanoflow liquid chromatography–mass spectrometry (nano-LC/MS), including contributions from our group, have largely overcome previous technical limitations associated with FFPE proteomics, enabling comprehensive molecular profiling of archived specimens [1,2,3,4,5,6,7,8,9,10]. Because FFPE samples are routinely collected with detailed clinicopathological information, they provide a unique opportunity to investigate tumor biology directly in clinically relevant patient materials.

Neuroblastoma (NB) is a rare pediatric solid tumor characterized by marked biological heterogeneity and poor prognosis in high-risk cases [11]. Despite extensive genomic analyses, few recurrent genetic alterations or clinically actionable targets have been identified [12]. Consequently, there is growing interest in proteomic analyses, which can provide complementary insights beyond genomics and capture functional aspects of tumor biology. In rare pediatric cancers such as NB, where the systematic collection of fresh or frozen tissues is often challenging, FFPE archives represent an invaluable alternative for large-scale molecular and translational studies directly linked to real-world clinical outcomes.

Although neuroblastoma cell lines are widely used experimental models, they incompletely recapitulate the structural and microenvironmental complexity of patient tumors [13]. Conversely, FFPE tumor tissues retain native tissue architecture and clinically relevant tumor-context information, making them a valuable resource for retrospective translational studies. However, how closely FFPE-derived proteomic profiles reflect or complement those obtained from conventional neuroblastoma cell-line models remains largely unknown. Clarifying the similarities and differences between these two widely used model systems is essential for interpreting retrospective proteomic findings in a clinically meaningful context. MYCN (N-myc proto-oncogene) is a well-established oncogenic driver in neuroblastoma, and its amplification is strongly associated with high-risk disease and aggressive clinical behavior [11,12]. MYCN regulates transcriptional programs related to cell cycle progression, metabolism, and ribosome biogenesis, thereby influencing global protein expression patterns. Accordingly, stratification according to MYCN status provides a biologically relevant framework for interpreting proteomic differences between samples.

High-risk NB typically exhibits substantial intratumoral heterogeneity, including mixed histological components with both undifferentiated and differentiated areas, as exemplified by ganglioneuroblastoma, nodular, in which morphologically divergent cell populations frequently coexist within the same lesion. As a result, conventional bulk tissue analyses often obscure tumor-intrinsic signals and fail to accurately reflect tumor-specific proteomic landscapes. LMD applied to FFPE sections enables pathologist-guided microdissection of morphologically defined tumor areas, thereby enhancing the purity and precision of proteomic characterization. This approach leverages routinely archived FFPE samples to yield robust molecular data that directly reflect tumor biology as observed in patients.

In this study, we performed comparative DIA-based proteomic profiling of laser-microdissected FFPE neuroblastoma tissues and established neuroblastoma cell lines. By systematically comparing patient-derived tumor tissues with in vitro models, we aimed to characterize their complementary biological features and evaluate the translational relevance of FFPE-derived proteomic data in pediatric oncology research.

## 2. Materials and Methods

### 2.1. Sample Collection

Seven formalin-fixed, paraffin-embedded (FFPE) samples were obtained from patients diagnosed with high-risk neuroblastoma between 2018 and 2023 at Kobe Children’s Hospital, Japan. All patients were classified as high-risk according to established clinical criteria. The cohort comprised five females and two males, with a median age of 4 years (range, 1–7 years). MYCN amplification status in FFPE samples was determined by fluorescence in situ hybridization (FISH) according to clinical diagnostic records. Tumor recurrence was observed in two cases. All patients were alive at the time of analysis. Clinical information was retrieved retrospectively from medical records. All procedures involving human tissue samples were approved by the institutional ethics committee of Kobe University Graduate School of Medicine (Kobe, Japan) (B230009).

Four human neuroblastoma cell lines were used for comparative analysis. CHP-134 and NB-69 were obtained from the RIKEN BioResource Research Center (Tsukuba, Japan). NH-12 was obtained from the Cell Resource Center for Biomedical Research, Tohoku University (Sendai, Japan), and SK-N-AS was obtained from the European Collection of Authenticated Cell Cultures (ECACC, Public Health England, UK). Cell lines were maintained under standard culture conditions as recommended by the respective repositories. In this study, relative MYCN mRNA expression levels in neuroblastoma cell lines were assessed by RT-qPCR using GAPDH as an internal control (Figure S1). This analysis was used to categorize the cell lines according to relative MYCN expression rather than for quantitative comparison of small differences in MYCN expression. CHP-134 and NH-12 showed markedly higher relative MYCN expression than SK-N-AS and NB-69. Accordingly, CHP-134 and NH-12 were classified as MYCN-high, whereas SK-N-AS and NB-69 were classified as MYCN-low.

### 2.2. Histopathological Assessment and Annotation of Tumor Regions

**Figure 1:**
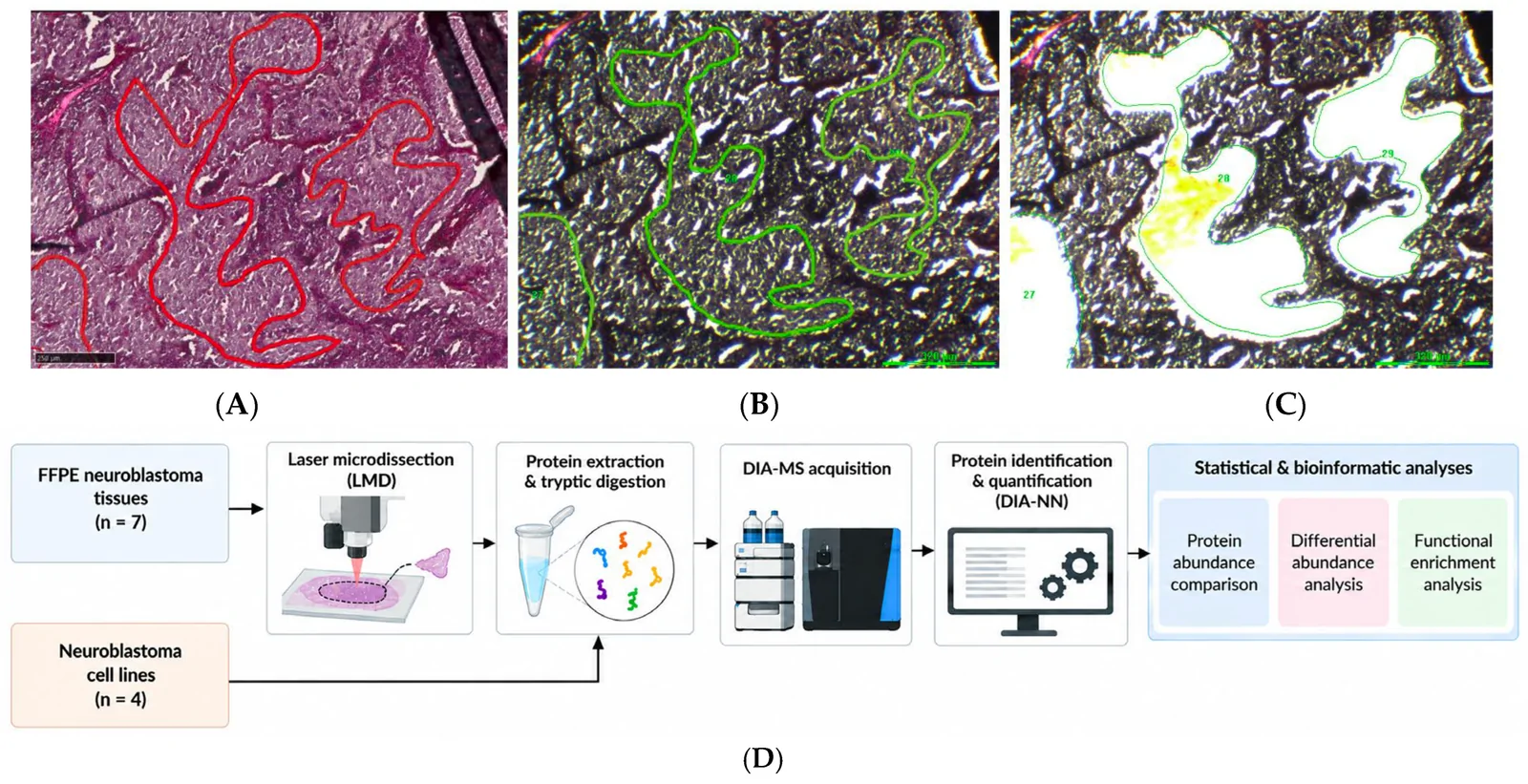
Histopathology-guided laser microdissection and overview of the experimental workflow. Representative workflow for identifying tumor regions and performing laser microdissection (LMD) of FFPE neuroblastoma tissue. (**A**) Tumor areas were precisely annotated on the digital H&E images. The corresponding regions were visualized (**B**) before and (**C**) after laser dissection, demonstrating accurate capture of annotated tumor regions and effective isolation of tumor-enriched areas for subsequent proteomic analysis. (**D**) Schematic overview of the proteomic and analytical workflow for FFPE neuroblastoma tissues and neuroblastoma cell lines, including LMD of FFPE tissues, protein extraction and tryptic digestion, DIA-MS acquisition, DIA-NN–based protein identification and quantification, and statistical and bioinformatic analyses.

Representative hematoxylin–eosin (H&E)–stained sections were reviewed by a board-certified pathologist to confirm tumor morphology and delineate viable tumor regions. Each slide prepared for laser microdissection (LMD) was immediately stained with H&E and digitized as a virtual slide using a NanoZoomer system (Hamamatsu Photonics, Hamamatsu, Japan). Tumor areas were carefully annotated on the digital images under pathological supervision (Figure 1A). These annotations served as the basis for subsequent LMD. To maximize tumor cell purity, vascular structures, necrotic regions, and non-neoplastic tissue components were excluded as much as technically feasible during region selection (Figure 1A–C).

### 2.3. Laser Microdissection, Protein Extraction, and DIA-LC–MS/MS Analysis

Laser capture microdissection (LMD) was performed using the Leica LMD7 system (Leica Microsystems GmbH, Wetzlar, Germany) as previously described [6,14]. Based on the annotated tumor regions, tumor-rich areas were selectively isolated from FFPE tissue sections mounted on membrane-coated Director Slides (AMR Inc., Tokyo, Japan). Microscope settings included ×6.3 and ×10 magnifications with a light intensity of 205. Laser settings were as follows: power 20, aperture 8, cutting speed 20, specimen balance 1, line spacing (Draw + Scan) 2, head current 90%, pulse frequency 2001 Hz, and offset 81. The cut shape was configured as “Draw + Scan” to ensure efficient and precise isolation of target regions. Representative dissected regions were documented as image files together with overview images of the entire tissue sections.

Tumor regions totaling 8 mm^2^, corresponding to approximately 15,000 tumor cells, were collected directly into the caps of 200 μL low-binding PCR tubes. The overall proteomic and analytical workflow for FFPE neuroblastoma tissues and neuroblastoma cell lines is summarized in Figure 1D. Proteins were extracted and digested with trypsin using the S-Trap™ system (Protifi, Farmingdale, NY, USA) as previously described [1,4,5]. Cell pellets from cultured neuroblastoma cell lines were processed using the same protocol. Briefly, harvested cells were lysed in an SDS-based buffer, and proteins were extracted and digested with trypsin using S-Trap™ mini spin columns.

The resulting tryptic peptides were analyzed in triplicate by nanoflow reverse-phase liquid chromatography coupled to an Orbitrap Fusion mass spectrometer (Thermo Fisher Scientific, San Jose, CA, USA) equipped with a nano-electrospray ionization source (AMR Inc., Tokyo, Japan). For cell-line samples, 5 μL of peptide digest (0.2 μg/μL; 1 μg peptides per injection) was injected for each LC–MS/MS analysis. For FFPE-LMD samples, peptide digests were prepared in a final volume of 20 μL, and 5 μL (25% of the total digest) was injected for each run. Because the peptide yield from laser-microdissected tissues was insufficient for accurate quantification, sample input was standardized by collecting identical tissue areas (8 mm^2^ from 5 μm-thick sections) for all FFPE specimens. Chromatographic separation was performed on an Advance UHPLC system (Bruker Daltonics, Bremen, Germany) coupled to an HTS PAL autosampler (CTC Analytics, Zwingen, Switzerland). Peptides were separated on an L-column2 ODS column (3 μm, 0.2 × 150 mm; CERI, Tokyo, Japan) using solvent A (0.1% formic acid in water) and solvent B (0.1% formic acid in acetonitrile). The gradient was programmed from 5% to 40% solvent B over 100 min, followed by column washing and re-equilibration, with a total run time of 120 min.

Data-independent acquisition (DIA) was performed under Xcalibur (version 4.4) control with a 3.0 s cycle time. Full MS scans were acquired in the Orbitrap at 60,000 resolution across *m*/*z* 495–745 with an AGC target of 2.0 × 10^5^ and a maximum injection time of 50 ms. DIA MS/MS scans were acquired at 30,000 resolution using staggered 4 *m*/*z* isolation windows with 1 *m*/*z* overlap across the precursor range *m*/*z* 498–742. Fragment ions were generated by higher-energy collisional dissociation (HCD) with a normalized collision energy of 30. Additional instrument parameters included an AGC target of 2.5 × 10^5^, maximum injection time of 100 ms, spray voltage of 1.8 kV, ion transfer tube temperature of 280 °C, and internal lock-mass calibration at *m*/*z* 391.28429 [15,16,17].

### 2.4. Proteomic Data Processing and Visualization

All FFPE tissue samples and neuroblastoma cell line samples were processed jointly in a single DIA-NN (v2.6.0) workflow for library-free data-independent acquisition proteomics using neural network–based peptide identification and interference correction [18] to ensure consistent peptide identification and quantification across sample types. Protein identification and quantification were performed in library-free mode against the UniProt human reference proteome database (https://www.uniprot.org/; accessed on 9 May 2024); reviewed entries with common contaminants). RT-dependent cross-run normalization and peak matching were enabled. Trypsin specificity was applied with up to two missed cleavages. Carbamidomethylation (C) was set as a fixed modification, whereas oxidation (M) and protein N-terminal acetylation were specified as variable modifications. A 1% false discovery rate (FDR) threshold was applied at both precursor and protein group levels. Only proteins identified with at least two distinct peptide sequences were retained.

Protein identification statistics and sample-specific protein analyses were initially performed using the complete set of identified proteins prior to application of abundance-based filtering. Proteins detected exclusively in FFPE tissues or neuroblastoma cell lines under the present analytical conditions were designated as FFPE-uniquely detected and cell line-uniquely detected proteins, respectively, and were used for descriptive comparisons only.

Technical replicate measurements were averaged to generate a single quantitative value for each biological sample. Technical-replicate reproducibility was assessed by calculating protein-wise coefficients of variation (CVs) from the raw protein intensities prior to replicate averaging. All downstream quantitative analyses, including principal component analysis (PCA), were performed at the biological-sample level (7 FFPE tissues and 4 neuroblastoma cell lines).

To ensure robust and comparable quantification, proteins detected in at least 70% of biological samples in both the FFPE group (≥5 of 7 samples) and the cell-line group (≥3 of 4 cell lines) were retained for downstream analyses. This threshold was selected to balance data completeness with quantitative robustness by retaining proteins reproducibly detected in the majority of biological samples while limiting the influence of extensive missingness on downstream statistical comparisons. Missing values were imputed after integration of all samples into a unified dataset using half of the minimum nonzero intensity observed for each protein across the biological samples. The filtered expression matrix was subsequently subjected to log_2_ transformation followed by quantile normalization.

After application of the biological-sample detection filter, 2186 proteins were retained for downstream analyses. Differential abundance analysis was performed using the limma package (version 3.64.1) in R (version 4.5.2). The unequal group sizes (7 FFPE samples and 4 cell lines) were retained in the analysis, and differential abundance was assessed at the biological-sample level without subsampling to equalize group sizes. Proteins with FDR < 0.05 and |log_2_ fold change| > 1 were considered significantly differentially abundant.

Functional enrichment analyses of significantly differentially abundant proteins were performed using the STRING database (version 12.0) via stringApp (version 2.2.0) in Cytoscape (version 3.10.3). Gene Ontology (GO) terms and pathway annotations were retrieved from STRING, and statistical significance was assessed using Benjamini–Hochberg adjusted *p*-values.

For visualization, Z-score normalization was applied across proteins. To assess data quality after normalization and missing value imputation, sample-wise protein intensity distributions and sample-to-sample correlation analyses were performed. For the group-level correlation analysis, the mean log_2_-transformed abundance of each protein was calculated separately across the seven FFPE samples and four cell lines, and Pearson correlation was calculated across the 2186 retained proteins. PCA was performed using the normalized biological-sample-level expression matrix to assess global sample separation. Hierarchical clustering heatmaps were generated using the 300 proteins with the highest variance across all samples solely for visualization of global expression patterns and reduction in low-variance noise, whereas all statistical differential abundance analyses were performed using the full filtered dataset consisting of 2186 proteins.

### 2.5. Flow Cytometric Assessment of Cell-Surface Protein Expression

To assess the concordance of selected cell-surface protein measurements between DIA-MS and an independent analytical method, CD44 and CD276 (B7-H3) expression was evaluated by flow cytometry in four neuroblastoma cell lines: SK-N-AS, NB-69, CHP-134, and NH-12. Cells were harvested and washed twice with phosphate-buffered saline (PBS; FUJIFILM Wako Pure Chemical Corporation, Osaka, Japan) containing 2% fetal bovine serum (FBS; Funakoshi Co., Ltd., Tokyo, Japan). Dead cells were excluded using Zombie Aqua Fixable Viability Kit (BioLegend, San Diego, CA, USA) according to the manufacturer’s instructions.

For surface staining, cells were incubated with PE-conjugated anti-human CD44 (clone IM7; BioLegend, San Diego, CA, USA) or PE-conjugated anti-human CD276 (B7-H3) (clone MIH42; BioLegend, San Diego, CA, USA) for 30 min at 4 °C in the dark. The corresponding PE-conjugated isotype controls were used to assess nonspecific staining. Rat IgG2b, κ isotype control (clone RTK4530; BioLegend) was used for CD44 staining, and mouse IgG1, κ isotype control (clone MOPC-21; BioLegend) was used for CD276 staining.

Following staining, cells were washed twice with PBS containing 2% FBS and analyzed using a BD LSRFortessa flow cytometer (BD Biosciences, San Jose, CA, USA). Viable cells were identified by exclusion of Zombie Aqua–positive events, and cell-surface protein expression was evaluated relative to the corresponding isotype controls. Flow cytometry data were analyzed using FlowJo software version 10.10.0 (BD Biosciences, Ashland, OR, USA), and representative histograms were generated for visualization.

## 3. Results

### 3.1. Sample-Specific Protein Identification

A total of 4511 proteins were identified from FFPE samples and 6812 from cell lines prior to application of the quantitative filtering criteria. The number of proteins identified in each individual sample is summarized in Supplementary Figure S2. Peptide identification depth was also lower in FFPE samples than in cell lines. Across individual FFPE samples, the median number of peptides identified per protein ranged from 2 to 3, compared with 4 to 5 in cell lines. The distribution of identified proteins according to peptide count further demonstrated a greater proportion of proteins supported by lower peptide numbers in FFPE samples (Table S2). This peptide-depth assessment was performed on the identification-level dataset prior to peptide-number filtering; proteins supported by fewer than two distinct peptide sequences were excluded before subsequent quantitative analyses, as described in the Methods.

**Figure 2:**
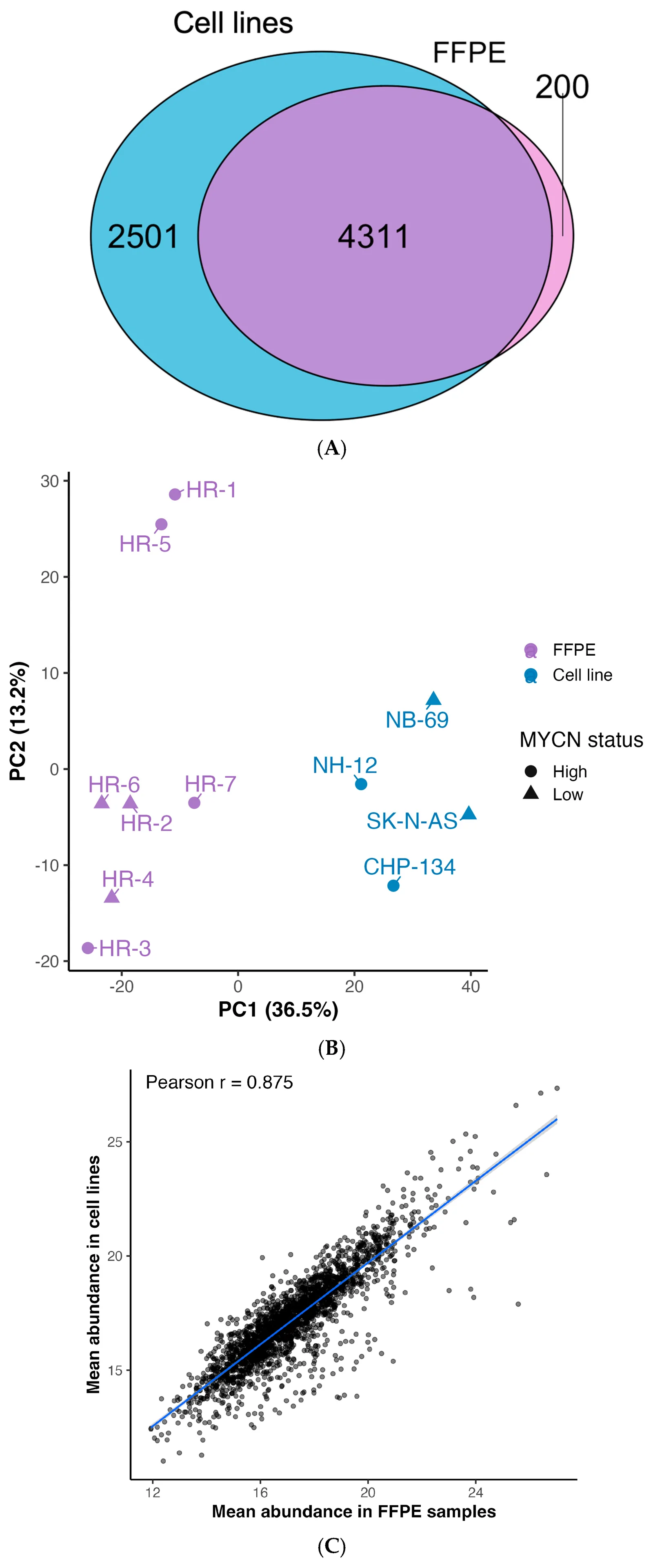
Global proteomic comparison between FFPE neuroblastoma tissues and neuroblastoma cell lines. (**A**) Venn diagram showing the overlap of proteins identified in FFPE tumor tissues and neuroblastoma cell lines. A total of 4511 proteins were identified in FFPE samples and 6812 in neuroblastoma cell lines, of which 4311 were shared between the two sample types. Additionally, 200 proteins were uniquely identified in FFPE tissues, whereas 2501 were uniquely identified in neuroblastoma cell lines. (**B**) Principal component analysis (PCA) based on the filtered quantitative proteomic dataset after averaging technical replicate measurements to the biological-sample level. FFPE tissues and neuroblastoma cell lines formed distinct clusters, primarily separated along PC1, indicating substantial differences in their global proteomic profiles. For FFPE samples, MYCN-high/low denotes MYCN amplification status determined by fluorescence in situ hybridization (FISH), whereas for cell lines it denotes relative MYCN mRNA expression determined by RT-qPCR. No clear clustering according to MYCN status was observed among the FFPE samples. The first two principal components accounted for 49.7% of the total variance (PC1, 36.5%; PC2, 13.2%). (**C**) Correlation analysis of protein abundance between FFPE tissues and neuroblastoma cell lines. Each dot represents a quantified protein plotted according to its mean log_2_-transformed abundance in FFPE tissues and cell lines. A strong positive correlation was observed between the group-mean protein abundance profiles (Pearson’s r = 0.875). This group-level correlation reflects similarity in average protein abundance across the quantified proteome and does not imply biological equivalence between the two sample types.

Among these, 4311 proteins were commonly detected in both sample types, whereas 200 proteins were detected exclusively in FFPE samples and 2501 proteins were detected exclusively in neuroblastoma cell lines (Figure 2A). This substantial overlap included canonical neuroblastoma-associated proteins such as NCAM1, DDC, and ISL1, supporting the notion that neuroblastoma cell lines retain a substantial proportion of the core neuroblastoma proteome.

The 200 FFPE-unique proteins included inflammatory mediators, coagulation-related proteins, and plasma proteins such as CRP, SAA1/SAA2, S100A8/A9, fibrinogen chains FGA and FGG, and complement components C1QB, C1QC, CFAB, and CFAH, which may reflect inflammatory responses and/or residual extracellular plasma-associated components. Importantly, FFPE-unique proteins also encompassed tissue-context–dependent neural and extracellular matrix–associated proteins, including DCC, NRXN2, SYN3, SCG3, CADM3, NEGR1, NCAN, FBN1, and DLK1. Together, these findings suggest that FFPE-derived proteomic profiles retain tissue-context–associated signals, including extracellular and neural components that are incompletely represented in cultured cells. However, some of these proteins may also originate from residual stromal, inflammatory, or plasma-associated components retained within the microdissected tissue regions. To further estimate residual non-tumor components in the microdissected FFPE samples, we compared the abundance ranks of representative neuroblastoma-associated and plasma-associated proteins. Neuroblastoma-associated proteins were consistently detected, including NCAM1 (7/7 samples; median abundance rank, 250), TH (6/7; rank, 171), and L1CAM (7/7; rank, 710). However, plasma-associated proteins were also detected at relatively high abundance, including ALB (7/7; rank, 16), FGB (7/7; rank, 142), C3 (7/7; rank, 290), FGG (7/7; rank, 307), and FGA (7/7; rank, 368) (Table S1). These findings confirm the retention of neuroblastoma-associated proteins while also indicating that residual plasma-associated components remained detectable after LMD.

By contrast, 2501 proteins were uniquely identified in neuroblastoma cell lines. These proteins included cell-cycle and mitotic regulators, such as CCNA2, PLK1, and AURKA, as well as metabolic and mitochondrial enzymes, including ACAD8, COQ5, HACL1, and IDH3B. Ribosomal and RNA-processing factors, including EIF1A, MPP10, and TSR3, were also preferentially detected, consistent with the high biosynthetic demand of rapidly proliferating cultured cells. In addition, oncogenic signaling molecules such as AKT1/2, ABL1/2, and RET, together with membrane-associated and trafficking-related proteins such as MMP14, were uniquely detected in cell lines. Collectively, these proteins may reflect, at least in part, the proliferative, biosynthetic, and metabolic characteristics of cultured neuroblastoma cells. However, the larger number of proteins detected in cell lines should not be attributed solely to biological differences. In addition to differences in sample complexity, formalin-induced protein cross-linking and differences in protein input, extraction efficiency, and peptide detectability may have contributed to the greater identification depth observed in cell-line samples. These sample-specific proteins were identified from the complete protein dataset prior to application of the 70% detection filter.

### 3.2. Quality Assessment of Proteomic Data

Principal component analysis (PCA) demonstrated clear global separation between FFPE neuroblastoma tissues and neuroblastoma cell lines (Figure 2B). PC1 accounted for 36.5% of the total variance and primarily distinguished clinical FFPE specimens from in vitro cell-line models, indicating substantial differences in their global proteomic landscapes. No clear clustering according to MYCN amplification status was observed among the FFPE samples.

Despite this separation, Pearson correlation analysis revealed a strong positive correlation in mean protein abundance across the quantified proteome between FFPE tissues and cell lines (r = 0.875; Figure 2C), indicating a substantial shared proteomic core across the two sample types. However, this group-level correlation reflects similarity in average protein abundance across the quantified proteome and does not imply biological equivalence between the two sample types.

Sample-to-sample Pearson correlation analysis further demonstrated high within-group similarity and clustering primarily according to sample type, supporting the overall consistency of the normalized quantitative proteomic dataset (Figure S3).

After quantile normalization, protein intensity distributions were highly comparable across all samples, indicating successful normalization and supporting downstream comparative analyses (Figure S4).

Protein-wise technical replicate CV analysis further supported the high analytical reproducibility of the DIA-MS workflow, with 84.7–97.8% of quantified proteins exhibiting technical CV values below 30% across biological samples.

### 3.3. Clustering Analysis of Shared Proteins

**Figure 3:**
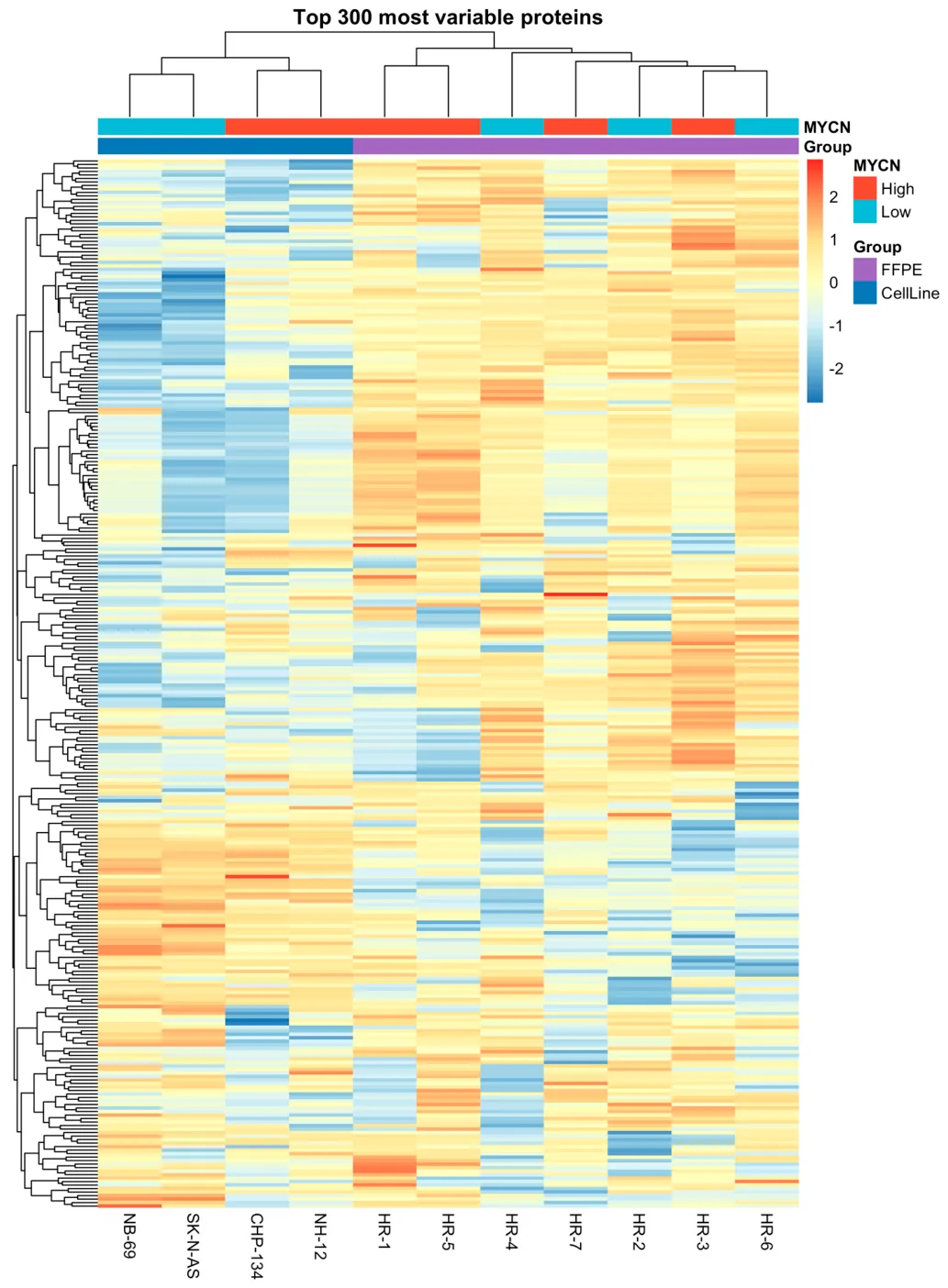
Hierarchical clustering heatmap of the top 300 most variable proteins among all biological samples. FFPE-derived neuroblastoma tissues and neuroblastoma cell lines formed clearly distinct clusters, reflecting divergent global proteomic profiles. Within the cell line cluster, the MYCN-high cell lines (NH-12 and CHP-134) clustered separately from MYCN-low cell lines (SK-N-AS and NB-69). In contrast, no clear clustering according to MYCN status was observed among the FFPE samples. For FFPE samples, MYCN-high/low denotes MYCN amplification status determined by FISH, whereas for cell lines it denotes relative MYCN mRNA expression determined by RT-qPCR.

Hierarchical clustering based on the 300 most variable proteins revealed a clear segregation between FFPE tumor samples and neuroblastoma cell lines (Figure 3). At the primary bifurcation of the dendrogram, all cell line samples branched apart from FFPE samples, underscoring the substantial differences in their global proteomic landscapes.

Within the cell line cohort, the two MYCN-high cell lines (CHP-134 and NH-12; Figure S1) clustered separately from the two MYCN-low cell lines (NB-69 and SK-N-AS). Given the limited number of cell lines examined, however, this observation should be considered exploratory. In contrast, no clear clustering according to MYCN status was observed among the FFPE samples.

### 3.4. Differential Protein Abundance Analysis

To further evaluate quantitative differences between FFPE tissues and neuroblastoma cell lines, differential abundance analysis was performed using the 2186 proteins retained after filtering. Proteins with FDR < 0.05 and |log_2_FC| > 1 were considered significantly differentially abundant.

Across the 2186 proteins retained for quantitative analysis, the median absolute log_2_ fold change between FFPE tissues and cell lines was 0.517 (IQR, 0.233–0.933). The median protein-wise variance was 0.358 (IQR, 0.175–0.752) in FFPE tissues and 0.164 (IQR, 0.072–0.392) in cell lines.

**Figure 4:**
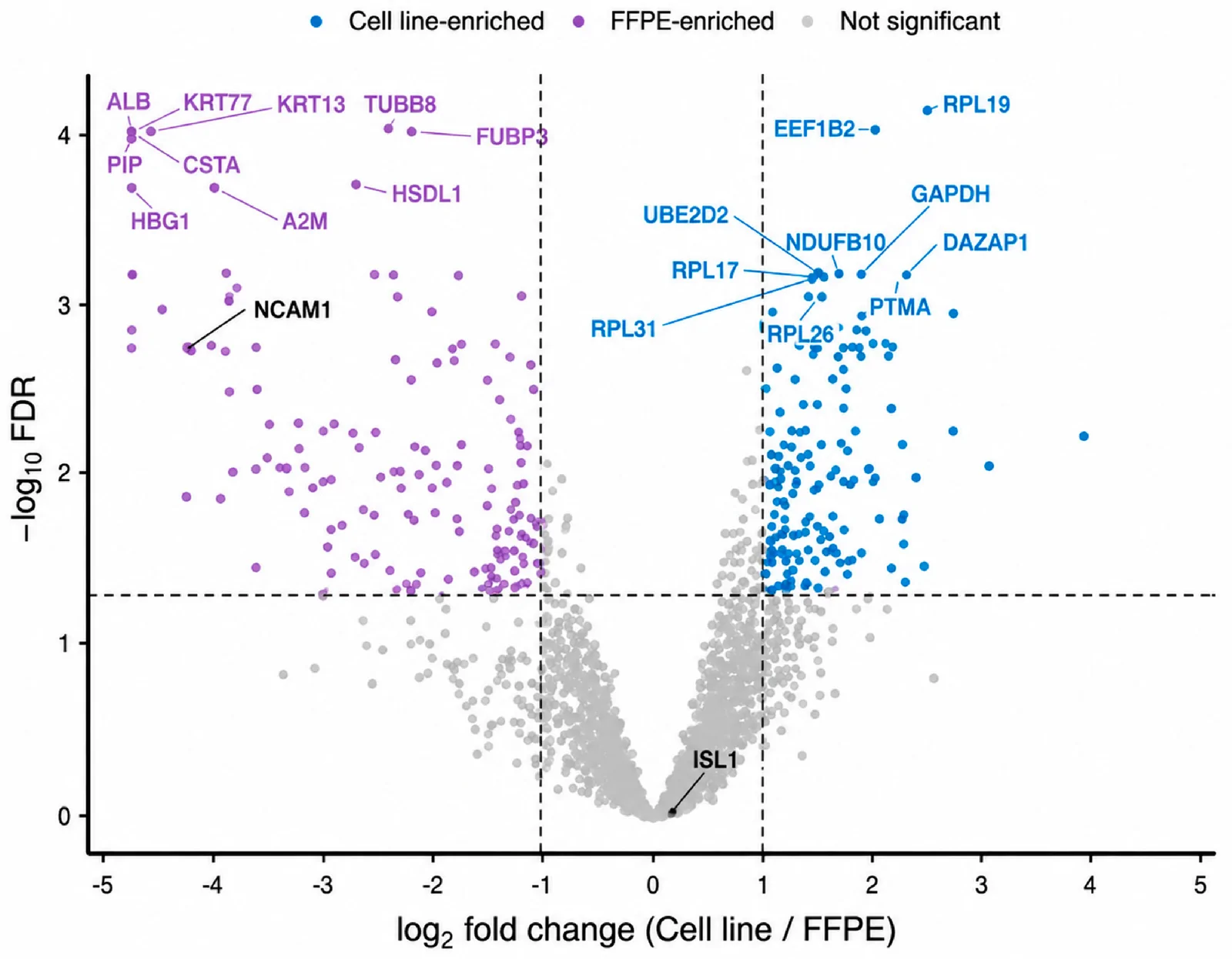
Differential protein abundance between FFPE neuroblastoma tissues and neuroblastoma cell lines. Volcano plot showing differential protein abundance between FFPE tissues and neuroblastoma cell lines. Differential abundance analysis was performed using the limma package. Proteins with a false discovery rate (FDR) < 0.05 and an absolute log_2_ fold change > 1 were considered significantly differentially abundant. Blue dots represent proteins enriched in cell lines, whereas purple dots represent proteins enriched in FFPE tissues. Gray dots indicate proteins that did not meet the significance criteria. Representative proteins with the highest statistical significance are labeled. Cell line–enriched proteins included ribosomal and mitochondrial components, whereas FFPE-enriched proteins were associated with tissue-specific and extracellular features. NCAM1 and ISL1 were additionally highlighted as representative neuroblastoma-associated proteins. DDC was not included in the volcano plot because it did not meet the criteria for inclusion in the final quantitative dataset.

Volcano plot analysis demonstrated clear segregation between proteins enriched in FFPE tissues and those enriched in neuroblastoma cell lines (Figure 4). A full heatmap including all 2186 filtered proteins is provided in Supplementary Figure S5. Compared with the heatmap of top 300 most variable proteins, this full heatmap includes proteins with lower expression variability and provides an overview of the entire filtered proteomic dataset.

Proteins enriched in cell lines were predominantly associated with intracellular biosynthetic and metabolic processes, whereas proteins enriched in FFPE tissues were mainly related to extracellular and structural components.

### 3.5. Functional Enrichment Analysis

**Figure 5:**
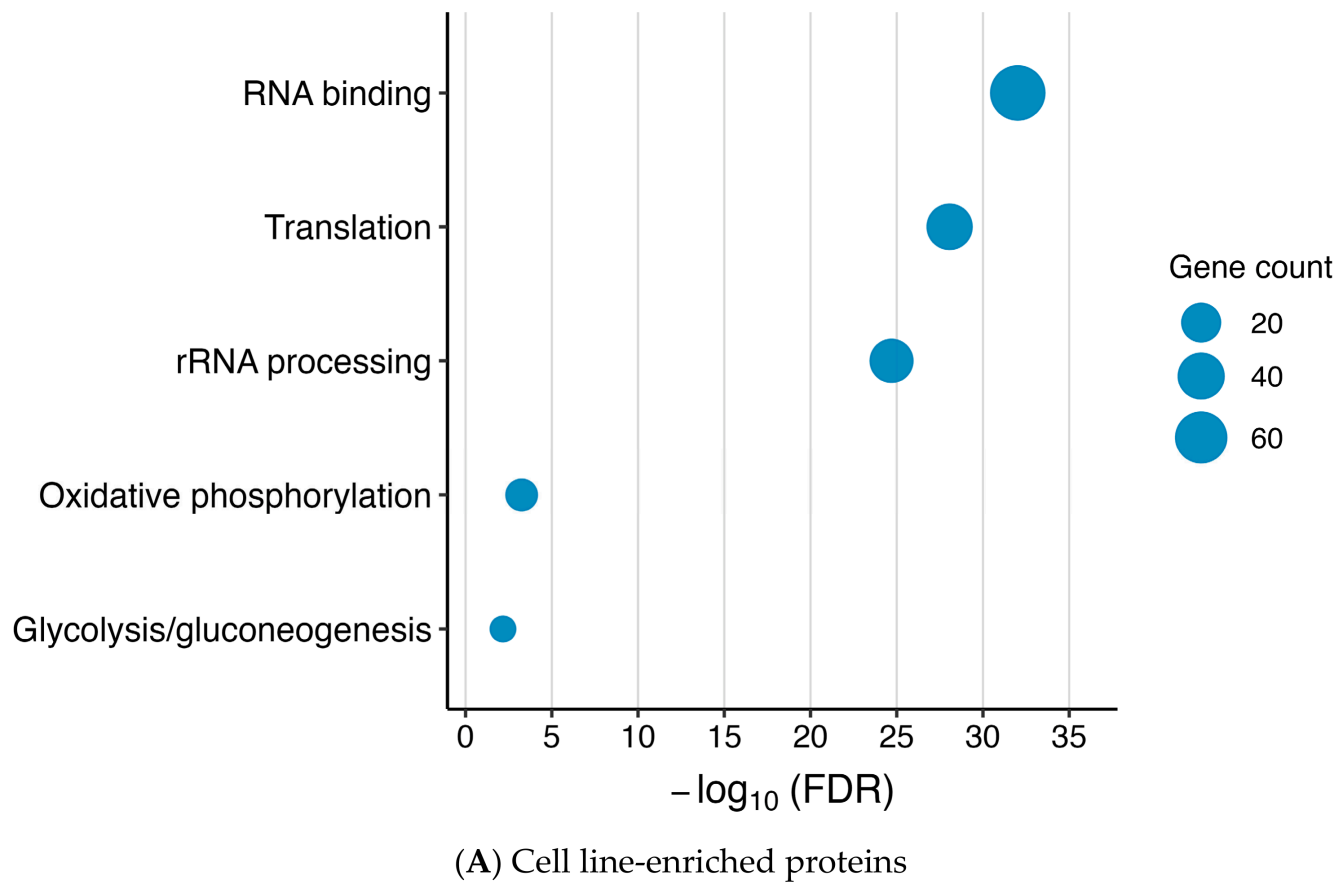

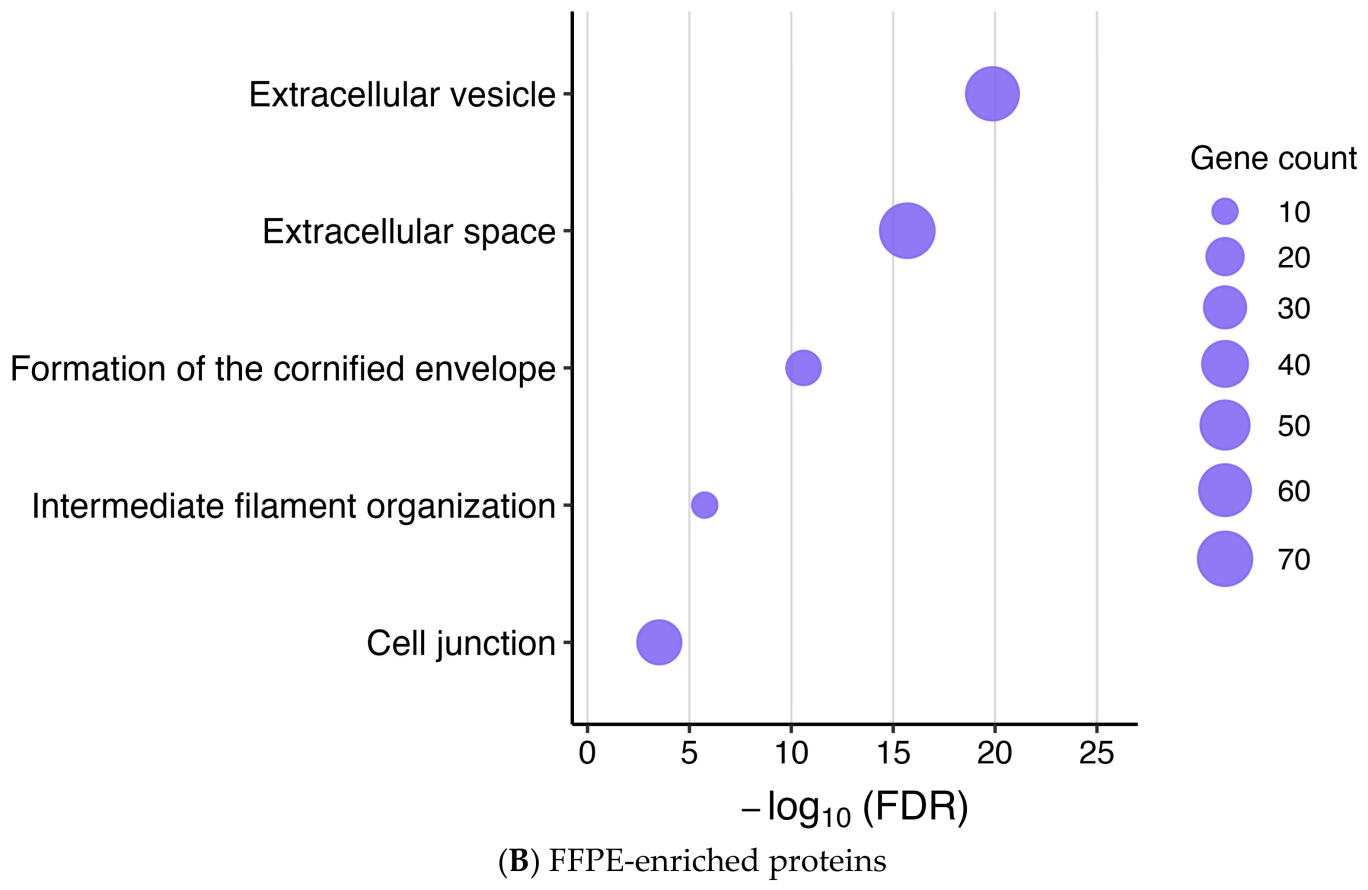
Functional enrichment analysis of proteins differentially abundant between FFPE neuroblastoma tissues and neuroblastoma cell lines. (**A**) Functional enrichment analysis of proteins significantly enriched in neuroblastoma cell lines. Enrichment analysis was performed using STRING based on proteins showing significantly higher abundance in cell lines than in FFPE tissues (FDR < 0.05, |log_2_ fold change| > 1). Enriched terms were primarily related to RNA binding, translation, rRNA processing, oxidative phosphorylation, and glycolysis/gluconeogenesis, consistent with the highly proliferative and metabolically active phenotype of cultured cells. The *x*-axis represents enrichment significance expressed as −log_10_(FDR), and bubble size represents the number of genes associated with each enriched term. (**B**) Functional enrichment analysis of proteins significantly enriched in FFPE tissues. Enrichment analysis was performed using STRING based on proteins showing significantly higher abundance in FFPE tissues than in cell lines (FDR < 0.05, |log_2_ fold change| > 1). Enriched terms were predominantly associated with extracellular vesicles, extracellular space, formation of the cornified envelope, intermediate filament organization, and cell junctions, consistent with preservation of tissue architecture and microenvironment-associated biological features. The *x*-axis represents enrichment significance expressed as −log_10_(FDR), and bubble size represents the number of genes associated with each enriched term.

To investigate the biological functions associated with differentially abundant proteins, Gene Ontology and pathway enrichment analyses were performed (Figure 5).

Proteins upregulated in neuroblastoma cell lines were strongly enriched in pathways related to intracellular biosynthetic and metabolic activity. Significant enrichment was observed for RNA binding, translation, rRNA processing, oxidative phosphorylation and glycolysis/gluconeogenesis (Figure 5A). These findings are consistent with the highly proliferative and metabolically active state of neuroblastoma cell lines under in vitro culture conditions.

In contrast, proteins enriched in FFPE tumor samples were predominantly associated with extracellular and tissue architecture–related processes, including extracellular vesicles, extracellular space, formation of the cornified envelope, intermediate filament organization and cell junctions (Figure 5B). These findings suggest preservation of extracellular components and tissue structural features within the native tumor microenvironment.

Notably, enrichment of intermediate filament organization and structural molecule activity further supports the retention of tissue architecture, while enrichment of extracellular vesicle– and extracellular space–associated proteins is consistent with preservation of microenvironment-associated features.

### 3.6. Comparison of Cell-Surface Protein Expression by DIA-MS and Flow Cytometry

**Figure 6:**
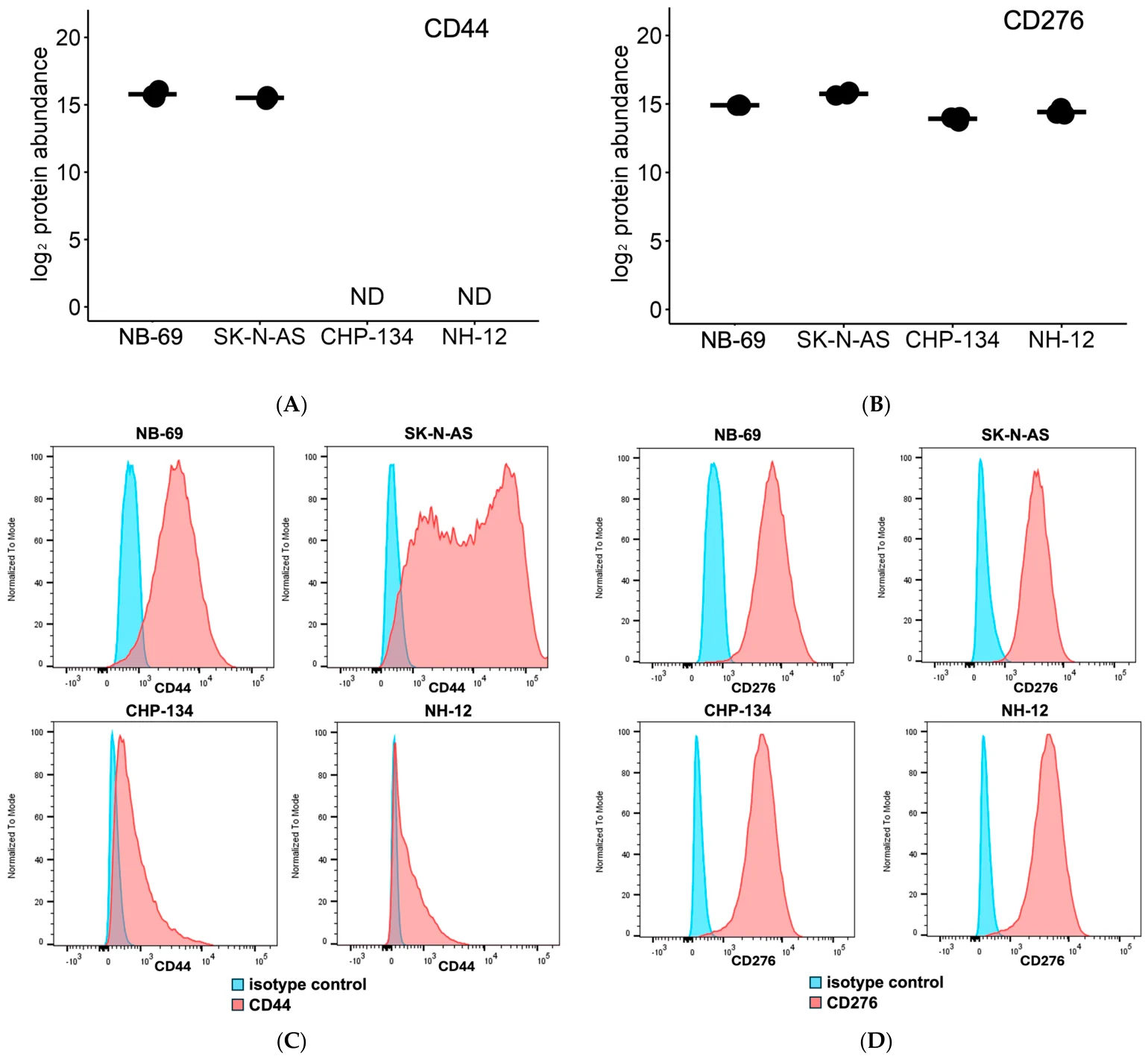
Comparison of CD44 and CD276 expression by proteomic and flow cytometric analyses. (**A**,**B**) LC–MS–based quantification of CD44 and CD276 protein abundance in four neuroblastoma cell lines. Data points represent technical replicates, and horizontal bars indicate mean values. ND, not detected. (**C**,**D**) Flow cytometric analysis of cell-surface CD44 and CD276 expression in the corresponding neuroblastoma cell lines. Red histograms represent CD44 and CD276 staining, and blue histograms indicate the corresponding isotype controls. Consistent with the proteomic findings, strong CD44 expression was observed in NB-69 and SK-N-AS cells, whereas CHP-134 and NH-12 cells showed minimal expression. CD276 was consistently detected in all four neuroblastoma cell lines by both LC–MS and flow cytometry, demonstrating concordance between the two analytical platforms.

The expression of two selected cell-surface proteins, CD44 and CD276, was compared between DIA-MS and flow cytometry in neuroblastoma cell lines. CD44 expression showed concordant patterns between the two methods, with high expression in NB-69 and SK-N-AS cells and minimal expression in CHP-134 and NH-12 cells (Figure 6A,C). CD276 was detected in all four cell lines by both LC–MS and flow cytometry (Figure 6B,D). These findings demonstrate concordance between DIA-MS and flow cytometry for the selected cell-surface protein expression patterns but do not constitute validation of the broader differential proteomic findings between FFPE tissues and cell lines.

## 4. Discussion

To our knowledge, this study presents the first direct comparison of proteomic profiles between laser-microdissected FFPE neuroblastoma tissues and established neuroblastoma cell lines using a DIA-based nano-LC/MS workflow. Although formalin-induced protein cross-linking has historically limited the application of FFPE materials in proteomic studies, recent methodological advances, including optimized SDS-based extraction and DIA-MS acquisition, now enable reliable and reproducible profiling from routinely archived clinical specimens [1,2,3,4,5,19,20]. By integrating these approaches with LMD, we demonstrated that high-quality proteomic data can be obtained from morphologically defined tumor regions, underscoring the value of FFPE-based proteomics for retrospective and translational cancer research.

Among the nonredundant 7012 proteins identified across all samples, 4311 were shared between FFPE tissues and cell lines, indicating a conserved proteomic core. This substantial overlap supports the notion that cell lines retain key neuroblastoma-associated molecular features. In contrast, proteins uniquely detected in FFPE samples included neural and extracellular matrix-associated proteins such as DCC, NRXN2, SYN3, SCG3, FBN1, and DLK1, together with inflammatory, coagulation, and plasma-associated proteins. These findings suggest that FFPE-derived profiles retain neural differentiation and tissue-context-associated features that are incompletely represented in cultured cells. Although laser microdissection was used to enrich tumor-cell–dominant regions, FFPE tumor tissues may retain tissue-context-associated components that are absent from cultured cell lines, which may contribute to the detection of inflammatory, extracellular and stromal proteins. In contrast, proteins uniquely detected in cell lines included cell-cycle regulators such as CCNA2, PLK1, and AURKA, metabolic and mitochondrial proteins including ACAD8, COQ5, HACL1, and IDH3B, and ribosomal and RNA-processing proteins such as EIF1A, MPP10, and TSR3. These findings are consistent with enhanced proliferative, biosynthetic, and metabolic activity in cultured neuroblastoma cells. Cultured neuroblastoma cell lines showed broader representation of membrane-trafficking and cell-surface proteins, supporting their utility for therapeutic-target and surfaceome-oriented studies. Previous surfaceome analyses using neuroblastoma cell lines have similarly demonstrated the value of in vitro models for identifying candidate neuronal cell-surface targets [21,22]. These profiles likely reflect adaptation to artificial growth conditions and continuous proliferation.

Functional enrichment analysis further demonstrated distinct biological characteristics of the two sample types. Cell lines were enriched in pathways related to RNA binding, translation, rRNA processing, oxidative phosphorylation, and glycolysis/gluconeogenesis, reflecting their highly proliferative and metabolically active phenotype under in vitro culture conditions. Regarding MYCN-associated patterns, FFPE tissues did not segregate according to MYCN status in either PCA or hierarchical clustering analyses. Given the limited number of FFPE cases analyzed, however, the absence of clear MYCN-associated clustering should not be interpreted as evidence for the absence of an MYCN-associated proteomic signature. The small sample size may have limited our ability to detect such a pattern, while additional biological heterogeneity related to the in vivo tumor microenvironment may also have contributed to the observed variability.

The differences between FFPE tissues and cell lines likely reflect a combination of biological context (in vivo tumor tissue versus cultured cells) and methodological factors inherent to the comparison of FFPE-LMD tissues with cultured cell lines. In particular, formalin fixation, differences in protein input and sample complexity, and differences in protein extraction and peptide detectability may have contributed to the observed proteomic differences. Therefore, the differential abundance patterns observed in this study should not be interpreted as exclusively reflecting intrinsic biological differences between neuroblastoma tissues and cell lines.

Consistent with these findings, both principal component analysis and unsupervised hierarchical clustering revealed clear separation between FFPE tissues and cell lines, indicating substantial differences in their global proteomic architectures. Within the cell-line cohort, the MYCN-high cell lines clustered separately from the MYCN-low cell lines, although this observation should be considered exploratory because only four cell lines were examined. These findings underscore the importance of interpreting molecular findings derived from in vitro systems within the broader context of patient-derived tissues.

The direct overlap of 4311 proteins supports the presence of a substantial shared proteomic core between FFPE tissues and cell lines. In addition, the group-mean protein abundance profiles were strongly correlated (r = 0.875), indicating broad similarity in average abundance across the quantified proteome. This correlation should nevertheless be interpreted together with the clear separation observed by PCA and the distinct differential-abundance and enrichment profiles. Flow cytometric analysis showed concordant expression patterns for CD44 and CD276 in the four neuroblastoma cell lines, providing an independent comparison of selected cell-surface protein measurements obtained by DIA-MS. These experiments were intended to assess the consistency of selected protein measurements across analytical platforms rather than to validate the broader FFPE-versus-cell-line differential proteomic or functional enrichment findings. Experimental validation of representative proteins from the major enriched pathways will be important in future studies.

Collectively, our results indicate that FFPE-based proteomics and cell-line models capture distinct but complementary aspects of neuroblastoma biology. While FFPE tissues retain tumor-specific differentiation states and microenvironmental contexts, cell lines preferentially reflect tumor-intrinsic programs under controlled experimental conditions. Integrating these two systems enables clinically grounded molecular observations to be functionally interrogated, thereby enhancing their translational relevance. This complementary framework may facilitate the prioritization of candidate biomarkers and therapeutic targets that are both biologically meaningful and clinically applicable.

The main limitation of this study is the relatively small number of FFPE cases analyzed. In addition, the cell-line cohort consisted of four established neuroblastoma cell lines and therefore does not fully capture the biological heterogeneity of neuroblastoma. Although technical replicate measurements were averaged prior to analysis to generate biological-sample–level quantification, additional independent cell-line models would further strengthen the generalizability of the findings. In addition, relative MYCN expression in the cell lines was assessed using GAPDH as a single reference gene. Although the analysis was used only to distinguish cell lines with markedly high versus low MYCN expression, the use of a single reference gene represents a limitation. Given the exploratory nature of this study, the effect-size and variance estimates obtained here may also inform prospective sample-size calculations for future validation studies. Another important limitation is that FFPE tissues and cultured cell lines differ not only in their biological context but also in fixation, sample preparation, protein input, and cellular complexity. Therefore, the biological and technical contributions to the observed proteomic differences cannot be completely separated in the present study. Furthermore, although LMD enabled enrichment of morphologically defined tumor regions, residual stromal, inflammatory, vascular, extracellular, and plasma-associated components may have contributed to the FFPE-derived proteomic profiles. Orthogonal validation was performed only in the cell-line models and not in the FFPE tissues; therefore, the flow-cytometric findings should not be interpreted as validation of the FFPE-versus-cell-line differential proteomic findings. However, given the technical challenges associated with FFPE-based proteomics and the rarity of high-risk pediatric tumors, our cohort provides a valuable proof of concept. Further orthogonal validation of additional candidate proteins, particularly in FFPE tumor tissues using approaches such as immunohistochemistry, will be important to confirm the proteomic findings. Future studies involving larger, multi-institutional FFPE cohorts with detailed clinical annotations will be essential to validate the generalizability of our findings and to explore their prognostic or predictive implications.

Taken together, our data demonstrate that FFPE-based proteomics is not only technically feasible but also biologically informative in neuroblastoma. Integrating patient-derived FFPE tissues with experimental cell-line models provides a complementary framework for biomarker discovery and therapeutic target prioritization, bridging clinically relevant observations with mechanistic investigation.

## 5. Conclusions

This study demonstrates that laser-microdissected FFPE neuroblastoma tissues analyzed using a DIA-based nano-LC/MS workflow provide biologically informative proteomic profiles that complement conventional neuroblastoma cell-line models. While cell lines predominantly reflect tumor-intrinsic proliferative, biosynthetic, and metabolic programs, FFPE tissues retain extracellular, structural, and microenvironment-associated biological features present in the native tumor context. Despite these systematic differences, a substantial shared proteomic core was retained between the two systems, supporting the continued utility of cell lines as experimental models when interpreted alongside patient-derived tissue data.

Furthermore, our findings highlight the translational potential of routinely archived FFPE specimens and support their complementary use with experimentally tractable cell-line models for retrospective biomarker discovery, molecular characterization, and therapeutic target prioritization in pediatric oncology.

## Data Availability

The proteomics dataset is not publicly available at this stage because it forms part of an ongoing research project. The raw DIA-MS files will be provided confidentially to the Academic Editor for verification.

## Supplementary Materials

The following supporting information can be downloaded at: https://www.mdpi.com/article/10.3390/biology15201839/s1, Figure S1: Relative MYCN mRNA expression levels in neuroblastoma cell lines; Figure S2: Number of proteins identified in individual FFPE and neuroblastoma cell-line samples; Figure S3: Sample-to-sample Pearson correlation analysis of the normalized quantitative proteomic dataset; Figure S4: Distribution of normalized protein intensities across biological samples; Figure S5: Unsupervised hierarchical clustering using all proteins retained after quality filtering; Table S1: Detection frequency and relative abundance ranks of representative neuroblastoma-associated and plasma-associated proteins in laser-microdissected FFPE neuroblastoma samples; Table S2: Peptide identification depth in individual FFPE neuroblastoma samples and neuroblastoma cell lines.

## Abbreviations

The following abbreviations are used in this manuscript:

ABL1/2	abelson murine leukemia viral oncogene homolog 1 and 2
ACAD8	acyl-CoA dehydrogenase family member 8
AKT1/2	AKT serine/threonine kinase 1 and 2
AURKA	aurora kinase A
C1QB/C	complement C1 q B/C chain
CADM3	cell adhesion molecule 3
CCNA2	cyclin A2
CFAB/H	complement factor B/H
COG	Children’s Oncology Group
COQ5	coenzyme Q5 methyltransferase
CRP	C-reactive protein
DCC	deleted in colorectal carcinoma
DDC	DOPA decarboxylase
DIA-MS	data-independent acquisition mass spectrometry
DIA-NN	data-independent acquisition by neural networks
DLK1	delta like non-canonical Notch ligand 1
EIF1A	eukaryotic translation initiation factor 1A
FBN1	fibrillin-1
FDR	false discovery rate
FFPE	formalin-fixed, paraffin-embedded
FGA/B/G	fibrinogen alpha, beta and gamma chain
H&E	hematoxylin and eosin
HACL1	2-hydroxyacyl-CoA lyase 1
IDH3B	isocitrate dehydrogenase 3 beta
ISL1	insulin gene enhancer protein 1
LMD	laser microdissection
MMP14	matrix metallopeptidase 14
MPP10	M-phase phosphoprotein 10
MS	mass spectrometry
MYCN	v-myc avian myelocytomatosis viral oncogene neuroblastoma derived homolog
NB	neuroblastoma
NCAM1	neural cell adhesion molecule 1
NCAN	neurocan
NEGR1	neuronal growth regulator 1
NRXN2	neurexin 2
PCR	polymerase chain reaction
PLK1	polo-like kinase 1
RET	rearranged during transfection
RNA	ribonucleic acid
S100A8/9	S100 calcium binding protein A8/9
SAA1/2	serum amyloid A1/2
SCG3	secretogranin III
SDS	sodium dodecyl sulfate
SYN3	synapsin III
TSR3	TSR3 ribosome biogenesis protein
