# Supplementary material for: Comparative DIA Proteomics of Neuroblastoma: Laser-Microdissected FFPE Tumors Versus Cell Lines
Source: Biology (Basel). 2026 Oct 14;15(20):1839. doi: 10.3390/biology15201839 (PMC13701799; doi:10.3390/biology15201839)
Supplement: Supplementary file 1 [file biology-15-01839-s001.zip › biology-4538647-supplementary.pdf]

Figure S1.

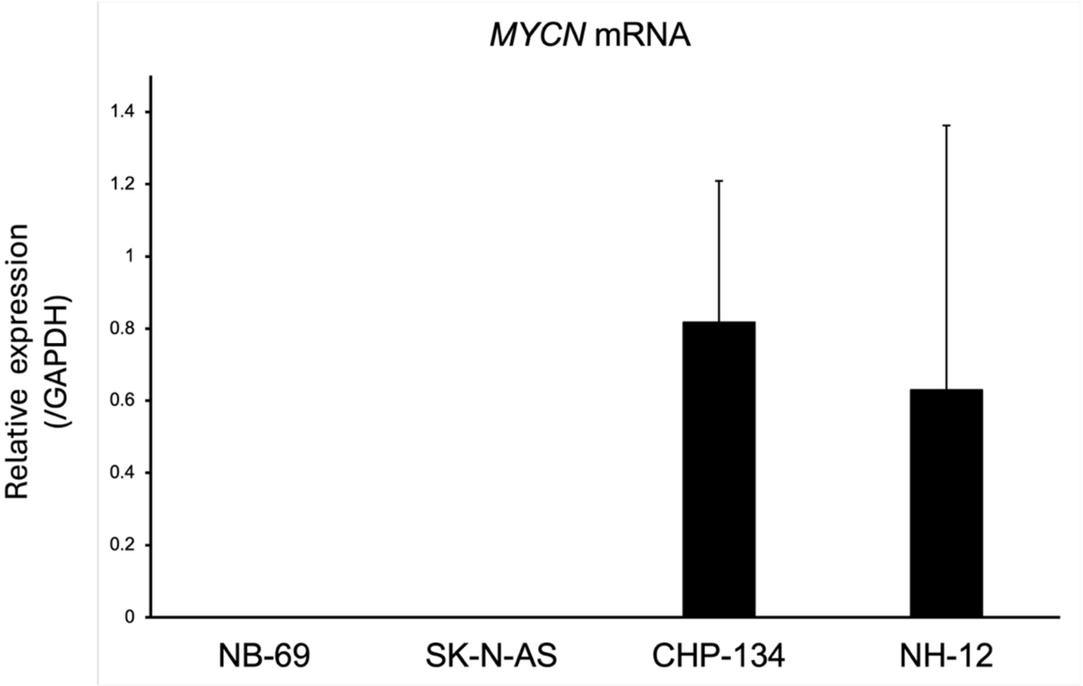

Figure S1. Relative MYCN mRNA expression levels in neuroblastoma cell lines. Relative MYCN mRNA expression levels were quantified by reverse transcription quantitative PCR and normalized to GAPDH expression. Bars represent the mean values of three technical replicates, and error bars indicate the standard deviation (SD). CHP-134 and NH-12 showed substantially higher MYCN expression levels than NB-69 and SK-N-AS, supporting their classification as MYCN-high and MYCN-low cell lines, respectively.

Figure S2

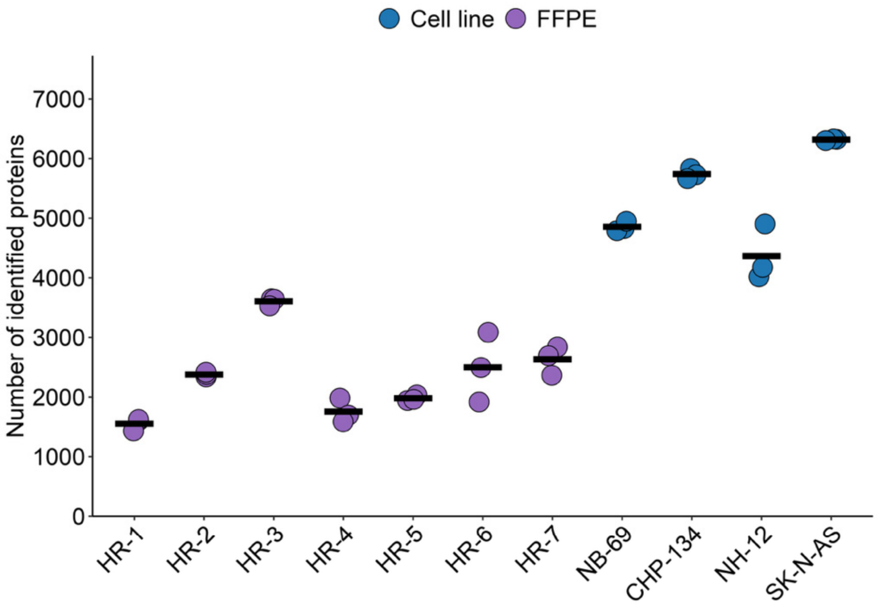

Sample	Replicate 1	Replicate 2	Replicate 3
HR-1	1603	1624	1432
HR-2	2338	2378	2416
HR-3	3648	3640	3530
HR-4	1983	1697	1587
HR-5	1942	2036	1961
HR-6	2495	1917	3086
HR-7	2839	2695	2365

Sample	Replicate 1	Replicate 2	Replicate 3
NB-69	4826	4790	4948
CHP-134	5832	5728	5664
NH-12	4176	4017	4903
SK-N-AS	6322	6329	6302

Figure S2. Number of proteins identified in individual FFPE and neuroblastoma cell-line samples.

Protein identifications obtained from each technical replicate are shown as individual dots, and horizontal bars indicate the mean number of identified proteins for each sample. The table below summarizes the protein identification counts for all technical replicates. Protein identification counts were generally consistent across technical replicates, although some variability was observed between repeated injections.

Figure S3.

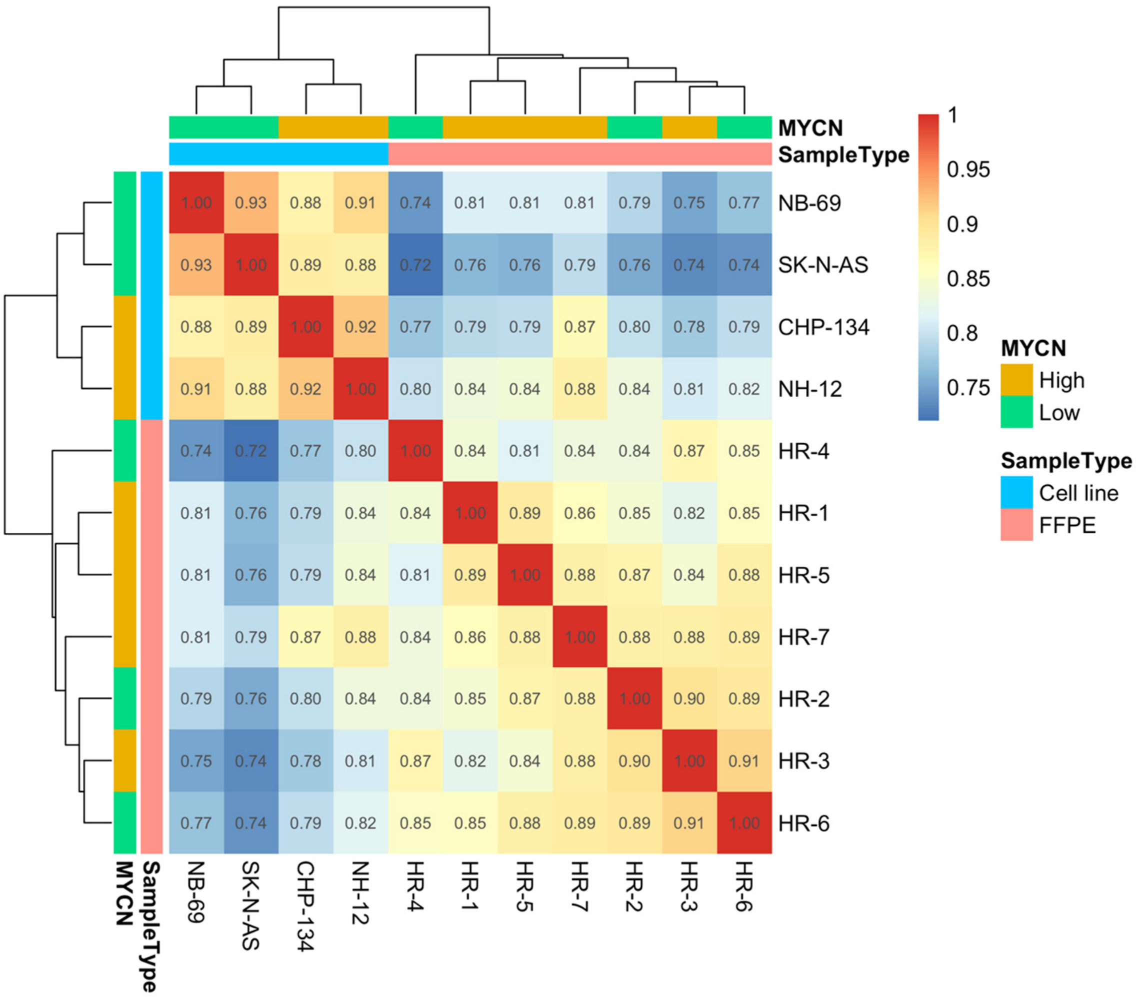

Figure S3. Sample-to-sample Pearson correlation analysis of the normalized quantitative proteomic dataset.

Pearson correlation coefficients were calculated using all 2,186 proteins retained after the 70% biological-sample detection filter following missing-value imputation and quantile normalization. Samples clustered primarily according to sample type, demonstrating high within-group similarity and clear separation between FFPE tissues and neuroblastoma cell lines.

Figure S4.

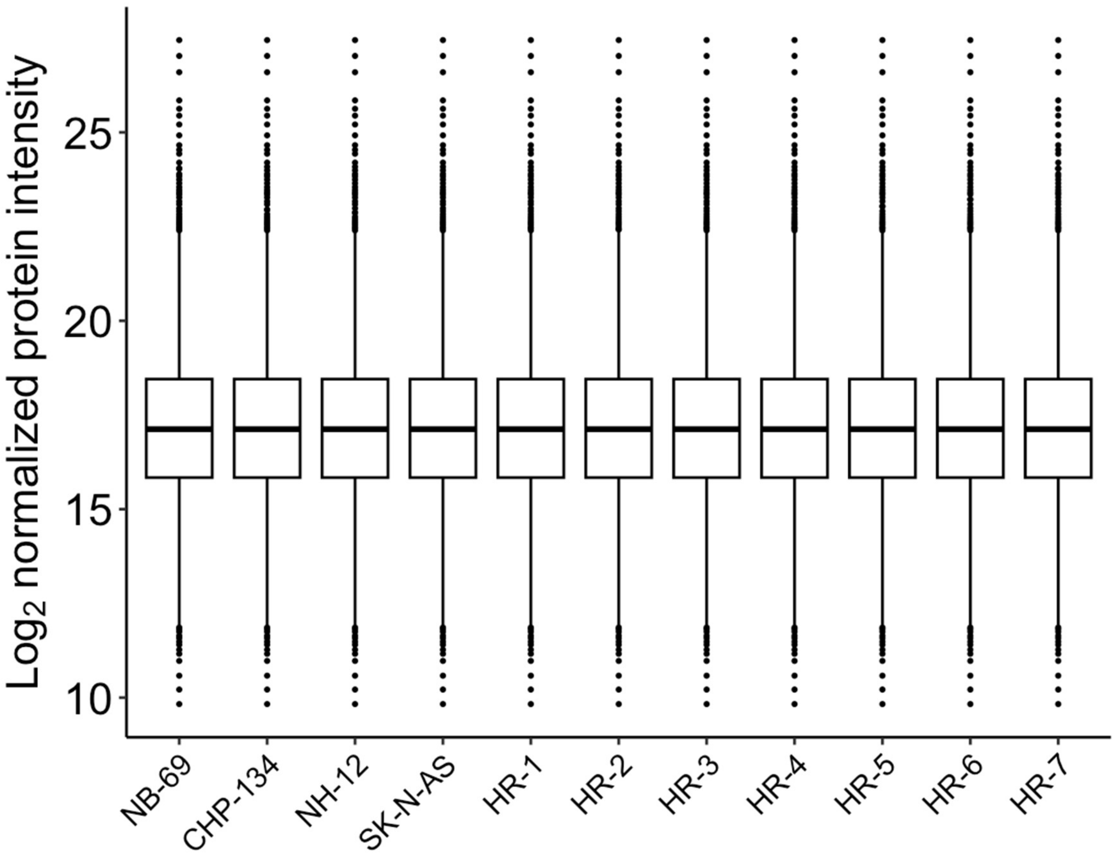

Figure S4. Distribution of normalized protein intensities across biological samples. Boxplots showing the distribution of protein abundances across biological samples using all proteins retained after the 70% detection filter. Missing values were imputed using half of the minimum nonzero intensity, followed by log2 transformation and quantile normalization. Similar intensity distributions across samples after normalization support the comparability of the quantitative proteomic data used for downstream analyses.

Figure S5.

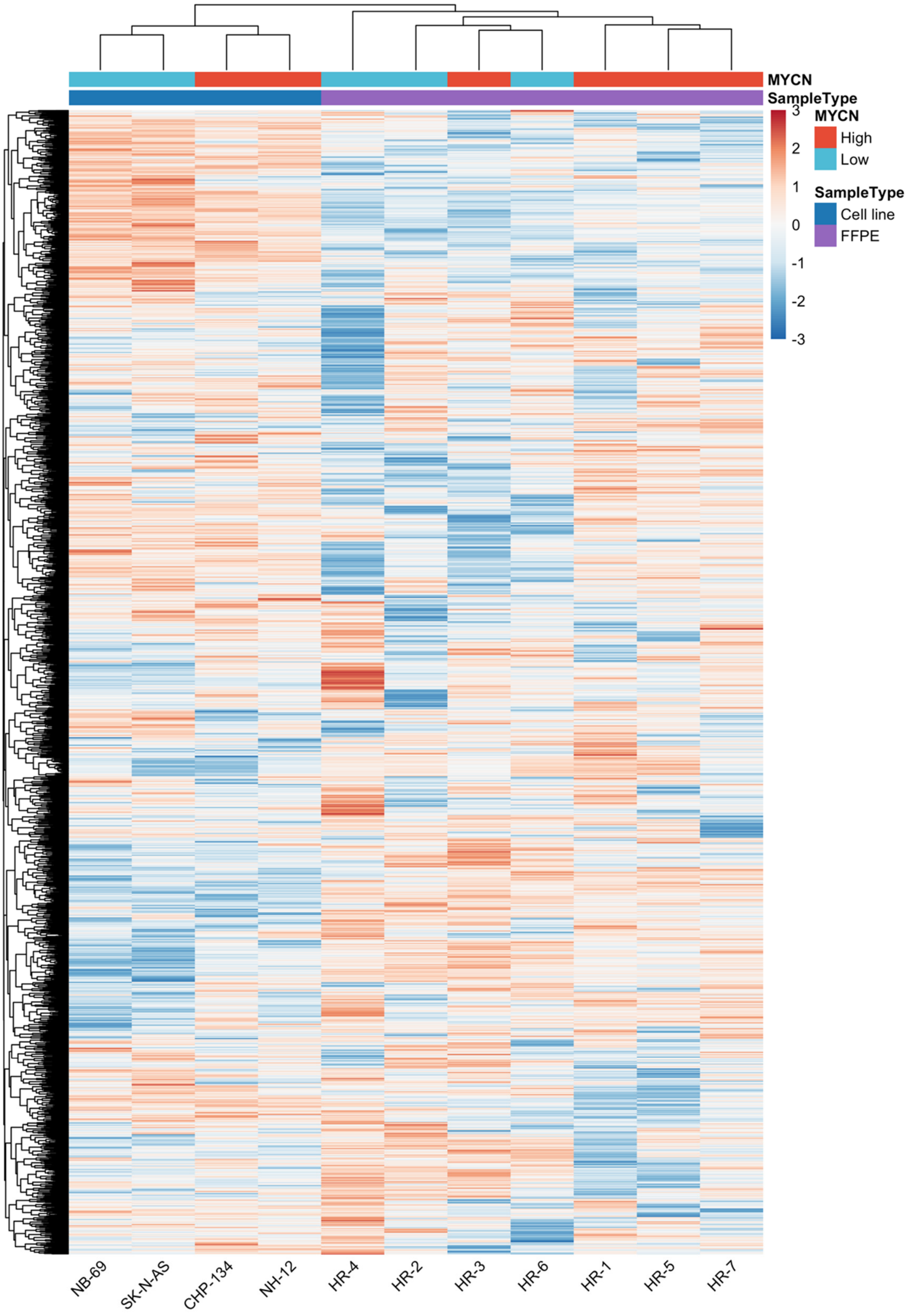
MYCN
SampleType
MYCN
High
Low
SampleType
Cell line
FFPE
3
2
1
0
-1
-2
-3
NB-69
SK-N-AS
CHP-134
NH-12
HR-4
HR-2
HR-3
HR-6
HR-1
HR-5
HR-7

Figure S5. Unsupervised hierarchical clustering using all proteins retained after quality filtering.

Heatmap showing unsupervised hierarchical clustering of all biological samples based on the complete proteomic dataset retained after the 70% detection filter. Protein abundances were imputed for missing values, log2-transformed, quantile normalized, and converted to row-wise Z-scores prior to clustering. Samples clustered primarily according to sample type (FFPE tissues versus neuroblastoma cell lines), consistent with the principal component analysis shown in Figure 2B. No clear segregation according to MYCN status was observed among the FFPE samples.

Table S1.

Category	Protein	Detection frequency	Abundance rank, median [IQR]	Abundance percentile, median [IQR]
Neuroblastoma-associated	Tyrosine hydroxylase (TH)	6/7	171 [144–336]	94.5 [83.3–95.2]
	Neural cell adhesion molecule 1 (NCAM1/CD56)	7/7	250 [160–268]	91.5 [90–94.1]
	L1 cell adhesion molecule (L1CAM/CD171)	7/7	710 [506–1124]	70.8 [52.1–83.9]
	DOPA decarboxylase (DDC)	4/7	1444 [1270–1788]	49.9 [34.3–61.7]
Plasma-associated	Albumin (ALB)	7/7	16 [14–18]	99.5 [99.3–99.5]
	Fibrinogen beta chain (FGB)	7/7	142 [108–210]	95.4 [91.9–96.3]
	Complement component 3 (C3)	7/7	290 [240–404]	90.3 [82–91.6]
	Fibrinogen gamma chain (FGG)	7/7	307 [210–406]	90.0 [84.0–93.0]
	Fibrinogen alpha chain (FGA)	7/7	368 [275–412]	86.9 [84.6–91.1]

Note: A lower abundance rank indicates higher relative protein abundance, with rank 1 representing the most abundant detected protein within each sample. Conversely, a higher abundance percentile indicates greater relative abundance. IQR, interquartile range.

Table S1. Detection frequency and relative abundance ranks of representative neuroblastoma-associated and plasma-associated proteins in laser-microdissected FFPE neuroblastoma samples.

To estimate the extent to which residual non-tumor components were retained in the microdissected FFPE samples, representative neuroblastoma-associated proteins (TH, NCAM1, L1CAM and DDC) and plasma-associated proteins (ALB, FGB, C3, FGG, and FGA) were evaluated across the seven FFPE samples. Detection frequency represents the number of FFPE samples in which each protein was detected. Relative abundance was assessed by protein abundance rank and percentile within each individual FFPE sample and is summarized as the median and interquartile range across the seven samples.

Table S2.

Sample	Peptides per protein, median [IQR]	Distribution of identified proteins by peptide count (%)				
		1 peptide	2 peptides	3-5 peptides	6-10 peptides	≥11 peptides
HR-1	2 [1–4]	36.8	20.4	24.9	12.1	5.8
HR-2	2 [1–4]	37.1	19.4	25.0	11.8	6.8
HR-3	3 [1–6]	29.7	18.4	26.5	15.7	9.6
HR-4	2 [1–4]	40.0	19.2	24.2	10.1	6.5
HR-5	2 [1–4]	36.6	19.3	25.9	11.8	6.3
HR-6	3 [1–5]	31.4	17.6	28.1	14.4	8.5
HR-7	2 [1–5]	33.4	17.9	27.3	13.7	7.7
NB-69	4 [2–8]	19.7	15.0	26.7	21.0	17.6
SK-N-AS	5 [3–11]	12.4	12.5	26.0	23.6	25.6
CHP-134	5 [2–10]	14.1	12.3	25.4	23.6	24.6
NH-12	4 [2–8]	21.6	14.7	27.3	20.5	15.8

Note: Percentages were calculated relative to the total number of proteins identified in each sample. IQR, interquartile range; FFPE, formalin-fixed, paraffin-embedded.

Table S2. Peptide identification depth in individual FFPE neuroblastoma samples and neuroblastoma cell lines.

Peptide identification depth was evaluated for each biological sample using peptide identifications combined across the three technical DIA-MS runs. For each sample, the number of peptides identified per protein is summarized as the median and interquartile range (IQR). Proteins were also categorized according to the number of peptides identified per protein (1, 2, 3–5, 6–10, or ≥11 peptides), and the proportion of identified proteins within each category was calculated. This assessment was performed before application of the ≥2-peptide criterion used for subsequent quantitative analyses.
